# Investigation and management of iron deficiency anaemia in a specialist palliative care setting and the role of intravenous iron: a descriptive analysis of hospice data

**DOI:** 10.12688/amrcopenres.12963.1

**Published:** 2021-01-28

**Authors:** Thomas Steele, Helen Bonwick, Amara Callistus Nwosu, Laura Chapman

**Affiliations:** 1 Marie Curie Hospice, Liverpool, Merseyside, L25 8QA, UK; 2 International Observatory on End of Life Care, Lancaster University, Lancaster, Lancashire, LA1 4YG, UK; 3 Liverpool University Hospitals NHS Trust, Liverpool, Merseyside, L7 8XP, UK

**Keywords:** Anaemia, iron deficiency, intravenous iron, hospice, palliative care

## Abstract

**Background:**

Anaemia is common in hospice populations and associated with significant symptom burden. Guidelines recommend investigating for and treating iron deficiency (ID), but there is little evidence of this practice in palliative care populations. This report describes the results of investigations for and subsequent management of ID in a UK hospice.

**Methods:**

This is a descriptive study of routine clinical data. Laboratory and clinical records were reviewed retrospectively for 12 months following the implementation, in August 2018, of routine investigation for ID amongst patients with clinically relevant anaemia in whom treatment would be considered. Absolute (AID) and functional iron deficiency (FID) were diagnosed using established definitions and treatments recorded.

**Results:**

Iron status was evaluated in 112 cases, representing 25/110 (22.7%) of those with mild, 26/76 (60.5%) moderate and 41/54 (75.9%) severe anaemia. Twenty-eight (25%) were defined as having AID, 48 (42.8%) FID and 36 (32%) no ID. There was a significant difference between groups in symptoms triggering haemoglobin check and diagnosis, with a higher proportion of patients with classic symptoms of anaemia and gastrointestinal malignancy in those with AID. Intravenous iron was given on 12 occasions in the hospice with no major adverse events. Subjective symptom benefit in 7 cases and a significant increase in overall mean haemoglobin were observed.

**Conclusions:**

This report describes the outcome of investigations for iron deficiency in patients with clinically significant anaemia in a UK hospice. Results indicate iron deficiency is common and can be safely treated with intravenous iron replacement, within current guidelines, in a hospice setting. Further research should define the optimum use of this approach in palliative care patients.

## Plain language summary

Many patients supported by hospices have anaemia. This can lead to symptoms including tiredness and shortness of breath. In severe cases, patients may be given a blood transfusion, however this carries significant risks and blood is a limited resource. Anaemia can be caused by a lack of iron. Iron deficiency can be treated with iron tablets or injections. However, iron tablets can have a lot of side effects and patients generally have to go to hospital for iron injections. There is little information into how important this is for anaemia that we see in hospices and how it should be treated.

We looked at the results of tests for iron deficiency done at a UK hospice in day-to-day practice. The hospice started doing these tests for appropriate patients with anaemia after it was recommended in recent guidelines. Out of the tests performed, a quarter showed iron deficiency. There were a higher number of patients with typical symptoms of anaemia and patients with a cancer of the gut in the group with iron deficiency. We also looked at treatment given for iron deficiency. Twelve were given iron injections at the hospice, which hasn’t been reported before. This study was not designed to investigate how well this worked but there were no major side effects. It also seemed to improve blood counts and help symptoms in most.

Overall, this study showed that we were able to test for iron deficiency at the hospice, we found it often and, in certain patients, were able to treat it with iron injections at the hospice. Research in the future should look at how we could best use this approach. It should also look at the effectiveness of iron injections in hospice patients.

## Introduction

Anaemia is common in hospice populations and associated with significant symptom burden ^1^. Red blood cell (RBC) transfusion is frequently performed, however benefit is often short-lived and risks, including reactions and circulatory overload, are significant, leading to calls for a more restrictive approach ^2– 4^. However, there is evidence that treating even mild to moderate anaemia may lead to symptomatic benefit and improvements in quality of life in patients with cancer ^5, 6^.

Assessing for and, if present, treating iron deficiency (ID) was a key recommendation from the recent UK comparative audit of blood transfusion practice in hospices and has the potential to treat symptoms of anaemia in certain patients without or in addition to transfusion ^4^. ID is known to be relatively common in patients with cancer and associated with advanced disease ^7^. Treatment of ID is recommended in oncology settings, with intravenous iron having evidence of quality of life benefit and superior efficacy and tolerability versus oral preparations ^8, 9^. Despite these considerations there is very little evidence related to ID in palliative care populations and neither investigating for, nor treating ID are routinely performed in palliative care settings ^4^.

In this report, we aim to describe the investigation and management of ID, including the use of intravenous iron, in a UK specialist palliative care unit in order to evaluate how national guidance translates to clinical practice.

## Methods

### Setting

Marie Curie Hospice Liverpool provides specialist palliative care for cancer and non-cancer patients in Liverpool, North West England. It has a 20 bedded inpatient unit as well as providing outpatient services and an ambulatory blood transfusion service that accepts direct referrals for patients with advanced cancer.

### Design

This is a retrospective, descriptive study of routine clinical data collected from August 2018-July 2019 following the implementation of guidance locally to investigate for ID in patients with clinically significant anaemia, in whom treatment would be appropriate. Prior to this, investigating for ID was not routine practice and to the knowledge of the clinical team there were no incidences of iron replacement being instigated locally by specialist palliative care services.

All full blood counts performed by the hospice were identified on the electronic laboratory record, with repeat sampling in the same patient excluded if performed within 6-weeks. Ferritin and iron study results were analysed if within 2 weeks of the index haemoglobin. Data on patient diagnosis, symptoms and treatments given for anaemia prior to and in response to the haemoglobin result were extracted from the electronic patient record. The presence of classic symptoms of anaemia at the time of haemoglobin check was defined as documented presence of one or more of fatigue, breathlessness or dizziness. As this was an analysis of anonymised routine clinical data generated for a locally approved audit, ethical approval was not required.

### Protocol for investigation of iron deficiency anaemia

Anaemia was defined as haemoglobin (Hb) below the age and sex-specific laboratory range and subsequently subclassified into mild (Hb >100g/L), moderate (Hb 80–100g/L) and severe (Hb<80g/L). Iron status was assessed in patients with anaemia deemed to be clinically significant, in whom oral or intravenous management would be appropriate in the context of their overall condition and prognosis. Ferritin was checked in all patients along with transferrin saturation (TSAT) in patients with malignancy ^9^. The tests were either retrospectively added to the index sample within a 48-hour period or taken at the time of haemoglobin check if anaemia was pre-existing or suspected. Blood samples were analysed according to standard practices at the local acute hospital.

### Protocol for interpretation of iron status and managing iron deficiency

**Figure 1.**
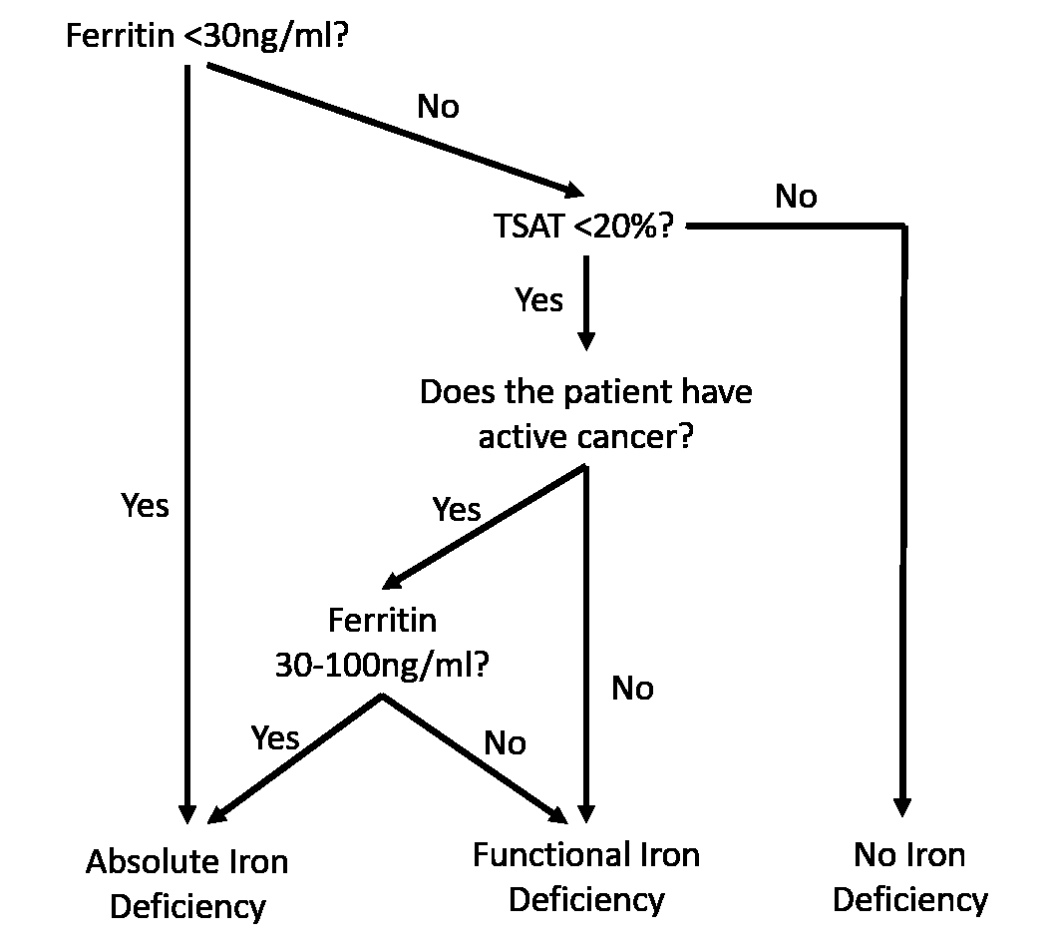
Algorithm for interpretation of ferritin and iron studies, based on general and oncology-specific guidance ^9, 10^.

The protocol used for interpreting iron status is summarised in Figure 1. Absolute ID was defined as ferritin <30ng/ml or, in patients with cancer, ferritin <100ng/ml and TSAT <20% ^8– 10^. Functional iron deficiency (FID) was defined as TSAT <20% and ferritin >30ng/ml or >100ng/ml in those with cancer ^8, 11^. Iron replacement was considered in those with absolute ID using these definitions, following national guidance to consider intravenous replacement when oral iron preparations are ineffective, not tolerated or contraindicated, or when there is a clinical need to deliver iron rapidly ^10^. If blood transfusion was deemed to also be indicated, this was given at least 24 hours prior to the iron replacement.

The protocol for delivery of intravenous iron was based on that of the partner NHS foundation trust providing pharmacy services (Liverpool Heart and Chest NHS Foundation Trust) and approved by the local medicines management and governance processes. Iron (III) isomaltoside (Monofer) was given as per the product literature with dosage calculated by weight and given as a single infusion. If the target dose exceeded the maximum single infusion dose (>20mg/kg) then the maximum dose was given in that infusion.

### Data analysis

Anonymised data was processed by Microsoft Excel and statistical analysis performed using SPSS v25 (IBM corp, USA). For comparisons between factors based on iron status, continuous data was assessed for normality and statistical tests employed as appropriate. For categorical data, chi squared test was used. Statistical significance was delineated at P<0.05.

## Results

### Iron status

From 1 ^st^ August 2018 to 31 ^st^ July 2019, excluding repeats within 6-weeks, haemoglobin was checked on 294 occasions across the hospice. In total, 240 (81.6%) had anaemia based on the laboratory reference range, 129 (44.2%) had Hb <100g/L and 54 (18.4%) Hb <80g/L. Blood transfusion was carried out in response to results in 59 cases.

Iron status was evaluated in 112 cases (57 inpatient, 55 outpatient). This represented 25/110 (22.7%) of those with mild anaemia, 46/76 (60.5%) of those with moderate anaemia and 41/54 (75.9%) of those with severe anaemia. A total of 101 patients (90.1%) had cancer, and 28 (25%) a malignancy of the gastrointestinal tract. The reason for haemoglobin check were typical symptoms of anaemia in 60 (53.6%), other symptoms in 28 (25%), external referral for transfusion in 21 (18.75%) and unclear in 3 (2.7%).

Overall, 28 (25%) were defined as having evidence of AID, 48 (42.9%) FID and 36 (32.1%) no ID. The clinical characteristics of patients with AID (potentially warranting treatment), FID and no evidence of ID are shown in Table 1. There were significant differences related to diagnosis and symptoms detected between groups, with a higher proportion of patients with classic symptoms and gastrointestinal malignancy in those with AID.

**Table 1.** Comparison of clinical characteristics depending on iron status.

Characteristic	Absolute Iron Deficiency (n=28)	Functional Iron Deficiency (n=48)	No Iron Deficiency (n=36)	P value
Age (mean; SD)	71.8 (66.9-76.8)	66.7 (63.1-70.2)	71.1 (67.4-74.8)	0.113 *^a^*
Sex (% female)	15 (53.6%)	20 (41.7%)	12 (33.3%)	0.266 *^b^*
Hb (mean; 95% CI)	84.1 (76.7-91.6)	87.5 (83.7-91.3)	93.1 (85.4-100.9)	0.136 *^a^*
MCV (mean 95% CI)	82.2 (78-86.4)	82.4 (80-84.7)	89.7 (87.3-92)	<0.001 *^a^*
Diagnosis *GI tract cancer* *Other cancer* *Non-malignant* *Missing*	14 (51.9%) 7 (25.9%) 6 (22.2%) 1	11 (22.9%) 37 (77.1%) 0 0	3 (8.6%) 27 (77.1%) 5 (14.3%) 1	<0.001 *^b^*
Symptoms prompting Hb check *Classic anaemia ^c^* *Other* *Transfusion service referral* *Missing*	18 (66.7%) 1 (3.7%) 8 (29.6%) 1	25 (53.2%) 14 (29.8%) 8 (17%) 0	17 (48.6%) 13 (37.1%) 5 (14.3%) 1	0.037 *^b^*
30 Day Mortality	3 (11.1%)	17 (35.4%)	7 (20%)	0.048 *^a^*

Statistical analysis: ^*a*^ ANOVA. ^*b*^Chi Square Test (excluding missing values). ^*c*^Classifed as fatigue, breathlessness, dizziness. Hb, haemoglobin.

### Management of iron deficiency and outcomes of intravenous iron

Intravenous iron was given on 14 occasions in 12 patients and oral iron replacement commenced in 6 patients. Of the remaining 10 patients with AID, 6 had clear decisions to not treat on clinical grounds documented and 4 no clear documentation. No patients treated with oral iron had subsequent intravenous iron. One did not tolerate the oral iron but no further treatment was appropriate, one had a stable haemoglobin and symptoms, and the remaining four had the oral iron stopped due to deteriorating condition, within 60 days of starting.

Twelve iron infusions were given at the hospice (in 10 patients) and two patients were referred for treatment elsewhere, one due to issues with intravenous access, the other due to patient preference. Of those delivered at the hospice, 4 (33.3%) were to inpatients, the remainder day cases. The clinical characteristics of the patients treated at the hospice are displayed in Table 2. There were no major adverse effects from iron infusion. One patient had minor extravasation of iron causing discolouration. Four patients (33.3%) received RBC transfusion for the same episode of anaemia. Three patients (25%) had subsequent RBC transfusion, between one and three months later. These had all had received RBC transfusion prior to the iron infusion and two were already classed as transfusion dependent, both showed an apparent decrease in transfusion dependency.

**Table 2.** Characteristics of patients who received intravenous iron infusion at Marie Curie Hospice Liverpool (n=10 patients and 12 infusions).

**Demographics (n=10)**	Female sex Age (median, range) Cancer diagnosis Gastrointestinal malignancy	6 (60%) 71 (35–88) 8 (80%) 6 (60%)
**Laboratory values** **(median, range)**	Haemoglobin (g/dL) MCV (fL) Ferritin (ug/L)	83.5 (55–114) 77.2 (68–98.5) 22 (5-80)
**Red blood cell transfusion**	Previously (within 3 months) For same episode	6 (50%) 4 (33%)
**Symptoms**	Fatigue Breathlessness Bleeding	12 (100%) 4 (33%) 2 (17%)
**Oral iron**	Taking, ineffective Not tolerated Contraindicated	6 (50%) 3 (25%) 3 (25%)

Of 12 patients receiving intravenous iron, 7 (58%) had a documented symptom benefit at four weeks, (including 6/8 of those who were given intravenous iron alone). Of the remaining five, one died within a week of the infusion, two did not have clinical follow up at the hospice and two did not have documented effect. Changes in haemoglobin for those with available results at 2–4, 6–8 and 10–14 weeks are shown in Table 3 and indicate a sustained mean increase. Those receiving subsequent transfusion were not included past the point of transfusion. Overall, 11/12 survived to 30 days, 11/12 to 60 days and 7/12 to 90 days.

**Table 3.** Changes in haemoglobin following intravenous iron infusion.

Follow up period	2–4 weeks	6–8 weeks	10–12 weeks
Number with available results Of all patients Simultaneous transfusion excluded	10 7	8 6	6 6
Missing data explanation Patient died Follow up elsewhere No available result in time-frame Results excluded due to transfusion	1 0 1 0	1 2 1 0	4 *^a^* 1 0 1 *^b^*
Haemoglobin (g/L) change from baseline (mean; 95% CI) All patients Simultaneous transfusions excluded	15.1(6-24.2) 15.1(2.6-27.7)	16.8(3.9-29.6) 18.7(1-36.3)	19.7(10.6-28.8) 19.7(10.6-28.8)
Proportion of patients with increase in haemoglobin (%) All patients Simultaneous transfusion excluded	9 (90%) 6 (85.7%)	7 (87.5%) 5 (83.3%)	6 (100%) 6 (100%)

Results from haemoglobin checks performed in usual clinical practice in the relevant time-frames. ^*a*^Five patients died within 12 weeks, however one also had follow up haemoglobin in 10–12 week timeframe. ^*b*^Three patients had subsequent transfusion, two of these also died so missing data classified under that explanation.

## Discussion

### Main findings

This descriptive study showed iron deficiency to be common among patients with clinically significant anaemia in this UK hospice population. ID appeared to be associated with gastrointestinal malignancy, which is consistent with reports in oncology settings ^7^. Rates were also higher amongst those in whom the original haemoglobin check was prompted by classic symptoms of anaemia or referral for transfusion compared with others, in whom anaemia may have been an incidental finding. The 30-day mortality appeared highest amongst those with FID, which is in keeping with the association of ferritin with inflammation and evidence of elevated ferritin being a poor prognostic indicator in several cancers ^12, 13^.

Importantly, the majority of patients with iron deficiency identified did receive specific treatment, including the novel use of intravenous iron in the hospice setting in 12 patients. This was well tolerated with no major adverse events and was managed safely both on the hospice ward and in an ambulatory setting. As this is a descriptive study, conclusions cannot be drawn regarding the efficacy of this intervention, however, apparent improvements in haemoglobin and symptoms were encouraging.

### Unique contribution and strengths

This project is the first to report the implementation of investigating for ID amongst patients with clinically significant anaemia into routine clinical practice in a hospice setting. The data generated is from a real-world setting meaning it is likely to be generalisable to similar settings. It is also the largest description of the iron status of patients in this setting with clinically significant anaemia and not exclusively receiving blood transfusion.

This is the first report to our knowledge of the use of intravenous iron in a hospice setting. This intervention was used in keeping with recommendations for treating ID and within the established indication of oral iron being ineffective, not tolerated or contraindicated ^10^. Intravenous iron is widely used in non-malignant conditions including renal and heart failure and evidence in cancer patients of superior efficacy, tolerability and speed of effect compared with oral iron supports it being considered for first-line use of intravenous iron in appropriate palliative care patients ^14– 16^.

### Comparison with previous work

Our findings confirm that anaemia in hospices is a significant problem. There is very little published data on management from either a technical or practical perspective. A recently published UK-wide audit described blood transfusion practice in hospices, which our report extends to patients with anaemia not receiving a transfusion ^4^. This audit suggested ID to be present in a significant proportion of patients, however, smaller, older case series in palliative care settings have shown conflicting reports of prevalence ^1, 4, 17^. ID may have been more common in our report as investigations were targeted at those with clinically significant anaemia.

Importantly, diagnosis of iron deficiency is not straight forward in palliative care settings and varying definitions have been used ^4, 18^. Ferritin levels are elevated in systemic inflammation, leading to a higher cut off being recommended for diagnosis of AID in cancer patients if TSAT is low ^8^. FID is a state in which there is insufficient iron available for erythropoiesis despite adequate total body iron stores and is important in the anaemia of malignancy ^11, 16^. Percentage hypochromic red blood cells has been proposed as an indicator of FID in palliative care patients but is not available widely, or locally ^18^. We used the combination of ferritin and TSAT, with specific interpretation in those with cancer to identify “absolute” and “functional” iron deficiency based on existing evidence, recommendations from oncology populations and local availability ^8– 11^. Our results support evidence that FID is very common in palliative care patients, however evidence to inform management of FID, particularly the role of intravenous iron, is significantly lacking ^18^.

### Implications to policy and/or practice

Our results suggest that guidance to investigate for and treat ID can be implemented into clinical practice in hospices. This allows treatment of anaemic patients with ID either in place of or addition to blood transfusion, or where transfusion would not be indicated but symptoms are still experienced. This is likely to be relevant across palliative care settings, even if local procedures and guidance varies.

Considering the appropriateness of investigations and treatments potentially close to the end of life is important. The facility to retrospectively add tests to existing blood samples helped ensure that patients did not undergo additional venepuncture. The data from patients receiving intravenous iron was encouraging and support the use of this intervention within its established indication in appropriately selected patients with AID in hospices. Although the majority of patients who received intravenous iron survived beyond 90 days, one died within 30 days. It is vital to consider overall prognosis, function and anticipated time to effectiveness when considering any form of management for anaemia. Oral iron supplementation is often associated with gastrointestinal side effects and decisions on prescription and continuation of this intervention close to the end of life should be individualised and the result of shared decision-making with the patient and those important to them. Utilisation of single doses of intravenous iron as a “first line option” may have the potential of reducing this burden and potentially facilitate deprescribing.

Investigating for and treating ID is associated with cost, however this may be offset by potential reduction of blood transfusion, unplanned hospital admission or other healthcare utilisation costs due to anaemia. Our ability to deliver this intervention via an established ambulatory transfusion service allowed it to be given to outpatients and the process was more straightforward than for blood transfusion as cross-matching is not required. Further research is needed to further address the cost effectiveness of this approach, effect on quality of life, and whether there is any role for intravenous iron in anaemia more widely, particularly FID.

### Limitations

This study is limited by its retrospective and descriptive nature. Investigating for ID was reliant of clinical decision making, therefore the prevalence in the wider hospice population cannot be inferred. Symptoms recorded were limited to the indication for the haemoglobin check and data was missing on ethnicity, functional status, co-morbidities and rationale for decisions over testing and treatment. The analysis of the patients receiving intravenous iron is also limited by the use of routine data which meant that there was no comparison group, follow up was not consistent in timing or assessment of symptoms. Furthermore, the number of patients receiving intravenous iron were small and we are unable to assess the effect of the overall approach on transfusion data at the hospice as service developments mean data would not be comparable over time.

## Conclusions

Investigating for iron deficiency and treating if present can be considered for appropriate hospice patients with clinically significant anaemia, particularly with underlying gastrointestinal malignancy and classic anaemia symptoms. In addition, we describe the safe use of intravenous iron in a hospice setting for the first time. Further research should focus on the optimum use of this intervention and detailed economic evaluation of this approach.

## Ethical approval

This analysis used routine clinical data in fully anonymised form that was generated from an audit of management of anaemia prompted by published recommendations from the national comparative audit of red cell transfusion practice in hospices ^4^. The audit and subsequent analysis were approved by the Marie Curie Hospice Liverpool audit governance process and additional ethical approval was not required.

## Data availability

### Underlying data

Figshare: Investigation and management of iron deficiency anaemia in a specialist palliative care setting and the role of intravenous iron: a descriptive analysis of hospice data, https://doi.org/10.6084/m9.figshare.13577882.v1 ^19^

Data are available under the terms of the Creative Commons Zero "No rights reserved" data waiver (CC0 1.0 Public domain dedication).
