# Supplementary material for: Investigation and management of iron deficiency anaemia in a specialist palliative care setting and the role of intravenous iron: a descriptive analysis of hospice data
Source: AMRC Open Res. 2021 Mar 22;3:6. Originally published 2021 Jan 28. [Version 2] doi: 10.12688/amrcopenres.12963.2 (PMC11064982; doi:10.12688/amrcopenres.12963.2)
Supplement: Peer review history

# Reviewer report: Reviewer response for version 2

**DOI:** 10.21956/amrcopenres.14052.r26654

**Published:** 2021-04-12

**Authors:** Edward A. Dickson

**Affiliations:** 1 National Institute for Health Research Biomedical Research Unit, Gastrointestinal and Liver Diseases, Nottingham University Hospitals NHS Trust, Nottingham, UK

I would like to thank Steele and colleagues for their response to the comments raised in the first version of their manuscript.

 All issues have been addressed well and the additional information provided adds further strength to the paper. Given the favourable outcome for many of their patients this work should help to generate plans for future prospective studies to address the important issue of iron deficiency anaemia in palliative care.

Is the work clearly and accurately presented and does it cite the current literature?

Yes

If applicable, is the statistical analysis and its interpretation appropriate?

Yes

Are all the source data underlying the results available to ensure full reproducibility?

Yes

Is the study design appropriate and is the work technically sound?

Yes

Are the conclusions drawn adequately supported by the results?

Yes

Are sufficient details of methods and analysis provided to allow replication by others?

Partly

Reviewer Expertise:

Clinical research fellow with an interest in iron deficiency anaemia and intravenous iron therapy in advanced cancer and palliative care.

I confirm that I have read this submission and believe that I have an appropriate level of expertise to confirm that it is of an acceptable scientific standard.

# Reviewer report: Reviewer response for version 1

**DOI:** 10.21956/amrcopenres.14034.r26625

**Published:** 2021-02-22

**Authors:** Edward A. Dickson

**Affiliations:** 1 National Institute for Health Research Biomedical Research Unit, Gastrointestinal and Liver Diseases, Nottingham University Hospitals NHS Trust, Nottingham, UK

Thank you for the opportunity to review this descriptive case series of anaemic patients being managed in a palliative care setting and the use of intravenous iron therapy in this group. Steele et al. make an important contribution to the literature in an area with limited evidence to date.

 The abstract describes the background and methods used by the authors well. However, the data in the results could be clearer. The percentages presented seem to differ to the results of the main manuscript and need to be checked. It could also be clearer that these percentages for iron studies relate to the total number of patients with confirmed anaemia. The authors state a “significant increase in overall mean haemoglobin were observed”. Does this mean clinically significant, or statistically significant?

 The plain English summary provides a good overview of the study but may benefit from a simplified explanation of anaemia for the lay person.

 The introduction is clearly written and highlights the issue being addressed well. References to the literature provide the rationale for the study and serve to summarise the key limitations of existing treatment options i.e. blood transfusion. The authors also describe the benefits of intravenous iron in other settings.

 The authors have described their methodology well with a definition of iron deficiency and a description of their treatment pathway. It would be useful to understand how clinically significant anaemia was defined – was this based on patient reported symptoms? Or clinician perception? The authors refer to the study as a locally approved audit and therefore it is important to state the standards against which they were auditing. They also describe implementation of local guidance before the study was conducted. It would be useful to know details of this for other centres to potentially employ.

 Division of anaemia into mild/moderate/severe is in keeping with WHO standards and would be useful to guide response to therapy. Particularly, as the authors acknowledge in the background, there may be benefits in treating even mild anaemia. The figure explaining how iron studies were interpreted is clear and well defined. The authors describe adding iron studies retrospectively to the full blood count. Was this performed by the clinical team at the time? Or was this prompted by the study team?

 Although this is a retrospective study the description of the data analysis would benefit from more detail on the outcome measures the authors planned to use. A description of the audit standards would also be welcome as mentioned earlier.

 The results section offers a good indication as to the prevalence of anaemia at this centre. It would be useful to see details on the 59 cases who were transfused. Were these patients more profoundly anaemic and deemed not appropriate for iron, or did they not receive iron despite meeting the local guidance for iron replacement therapy? Also, were any patient receiving myelosuppressive chemotherapy? This would be helpful to know given its confounding effect on haemoglobin. It appears all patients who received intravenous iron had absolute iron deficiency. Was there a local decision not treat functional iron deficiency with IV iron or was this coincidence? There is evidence to support its benefits in FID in other pathologies.

 The outcomes of intravenous iron therapy offer some important insights as to the feasibility of this treatment within the palliative care population. Did the two patients who received two doses require a split dose (due to the 20mg/kg/week limit) or were they given a further iron infusion at a later date as is sometimes used in other pathologies? Can the authors comment on why three patients had a subsequent RBC infusion after their iron. Was this due to a fall in haemoglobin, or a lack of benefit?

 Symptom relief is an important outcome measure for this patient group. Whilst I appreciate the authors are reliant on retrospective data it would be useful to know further details on which symptoms had improved if this is available.

 The data presented in Table 3 is helpfully divided by those who did and did not receive simultaneous blood transfusion. It is interesting to see that haemoglobin was higher in the non-transfused group at 6-8 weeks. Can the authors comment on this?

 The discussion is well structured and covers a number of important points. The limitations of the study are well acknowledged with good reference to previous smaller case series. The authors have demonstrated that their model is feasible and may offer patients an alternative option to repeated transfusion, particularly within an ambulatory setting. Steele et al. should be commended on publishing this case series in an area lacking high quality evidence. I would encourage the authors and other palliative care groups to publish future prospective data offering a robust evaluation of the efficacy of intravenous iron against a comparator group..

Is the work clearly and accurately presented and does it cite the current literature?

Yes

If applicable, is the statistical analysis and its interpretation appropriate?

Yes

Are all the source data underlying the results available to ensure full reproducibility?

Yes

Is the study design appropriate and is the work technically sound?

Yes

Are the conclusions drawn adequately supported by the results?

Yes

Are sufficient details of methods and analysis provided to allow replication by others?

Partly

Reviewer Expertise:

Clinical research fellow with an interest in iron deficiency anaemia and intravenous iron therapy in advanced cancer and palliative care.

I confirm that I have read this submission and believe that I have an appropriate level of expertise to confirm that it is of an acceptable scientific standard, however I have significant reservations, as outlined above.

# Response

**Published:** 2021-02-25

**Authors:** Thomas Steele

**Affiliations:** Marie Curie Cancer Care, UK

Thank you for reviewing our article, your positive feedback and constructive criticisms. We are glad to share our experiences with this approach to managing anaemia in a hospice setting, especially with there being so little evidence relating to non-transfusion therapies for anaemia in palliative care populations. We agree with your analysis of the limitations of our work and share your hope it gives encouragement towards trials of intravenous iron in this setting. As a result of your feedback we have made amendments to the paper and feel it improved as a result. I have responded to your comments point by point below.

***Thank you for the opportunity to review this descriptive case series of anaemic patients being managed in a palliative care setting and the use of intravenous iron therapy in this group. Steele et al. make an important contribution to the literature in an area with limited evidence to date.***

***The abstract describes the background and methods used by the authors well. However, the data in the results could be clearer. The percentages presented seem to differ to the results of the main manuscript and need to be checked. It could also be clearer that these percentages for iron studies relate to the total number of patients with confirmed anaemia. The authors state a “significant increase in overall mean haemoglobin were observed”. Does this mean clinically significant, or statistically significant?***

 Thank you for noting these issues in the results section of the abstract. A typo has been corrected relating to the proportion of those with moderate anaemia and we have clarified significant to refer to statistically (although this level of increase would generally also be felt to be clinically relevant).

***The plain English summary provides a good overview of the study but may benefit from a simplified explanation of anaemia for the lay person.***

 Thank you for this feedback we have added this.

***The introduction is clearly written and highlights the issue being addressed well. References to the literature provide the rationale for the study and serve to summarise the key limitations of existing treatment options i.e. blood transfusion. The authors also describe the benefits of intravenous iron in other settings.***

***The authors have described their methodology well with a definition of iron deficiency and a description of their treatment pathway. It would be useful to understand how clinically significant anaemia was defined – was this based on patient reported symptoms? Or clinician perception? The authors refer to the study as a locally approved audit and therefore it is important to state the standards against which they were auditing. They also describe implementation of local guidance before the study was conducted. It would be useful to know details of this for other centres to potentially employ.***

 We have amended our methods section significantly to clarify these points. Most significantly, the aim of the audit which generated the data is now described and the prompt for this, based on published national recommendations for practice, explained. A main component of the guidance was the protocols for investigation and management described in the methods section, this is now more clearly stated in the text.

***Division of anaemia into mild/moderate/severe is in keeping with WHO standards and would be useful to guide response to therapy. Particularly, as the authors acknowledge in the background, there may be benefits in treating even mild anaemia. The figure explaining how iron studies were interpreted is clear and well defined. The authors describe adding iron studies retrospectively to the full blood count. Was this performed by the clinical team at the time? Or was this prompted by the study team?***

 This was performed by the clinical team at the time and was all routine data. This is now clarified in the text. Unfortunately, it is not possible to tell how often there were “add ons” but we felt it important to emphasise the approach does not necessarily lead to more venepuncture for patients.

***Although this is a retrospective study the description of the data analysis would benefit from more detail on the outcome measures the authors planned to use. A description of the audit standards would also be welcome as mentioned earlier.***

 We have now added to the data analysis paragraph the planned primary and additional outcome measures, with context given by the description of the aim of the audit and specific standard relating to consideration of treatment of iron deficiency. We hope this gives a clearer picture to the reader of the data generated and how retrospective analysis was planned.

***The results section offers a good indication as to the prevalence of anaemia at this centre. It would be useful to see details on the 59 cases who were transfused. Were these patients more profoundly anaemic and deemed not appropriate for iron, or did they not receive iron despite meeting the local guidance for iron replacement therapy?***

 The decision-making process for considering iron was the same whether or not RBC transfusion was given- ie based on iron status (AID) rather what their heamoglobin was. In those with AID but transfusion potentially also appropriate the decision which to prioritise was based on clinical grounds, often in quite complex cases with multiple considerations (prognosis, severity of symptoms, suspected other aetiologies) which is unfortunately difficult to capture. We have added more details of the iron status specifically of those who were transfused which hopefully will help with context.

***Also, were any patient receiving myelosuppressive chemotherapy? This would be helpful to know given its confounding effect on haemoglobin. It appears all patients who received intravenous iron had absolute iron deficiency. Was there a local decision not treat functional iron deficiency with IV iron or was this coincidence? There is evidence to support its benefits in FID in other pathologies.***

 We only considered iron replacement in those meeting the definition of absolute iron deficiency. Although there is evidence supporting treatment of FID in certain conditions we lack evidence this is applicable across our general population, therefore treatment followed general national guidance. Note that the definition of AID used in cancer patients was adapted and not based on low ferritin alone to account for the presence and effect of inflammation in this situation.  Hopefully our results showing implementation of IV iron in those with this definition of AID demonstrate that specifically investigating IV iron in those with FID in palliative care settings is needed.

***The outcomes of intravenous iron therapy offer some important insights as to the feasibility of this treatment within the palliative care population. Did the two patients who received two doses require a split dose (due to the 20mg/kg/week limit) or were they given a further iron infusion at a later date as is sometimes used in other pathologies? Can the authors comment on why three patients had a subsequent RBC infusion after their iron. Was this due to a fall in haemoglobin, or a lack of benefit?***

 We have now specified when more than one dose was given these were separate episodes. If requiring over 20mg/kg the policy was to give maximum single dose and then review clinically as this was felt to be the best balance of benefit vs burden in our population. With regards to subsequent transfusion this is challenging to answer and it is not feasible to go deeply into individual level data in the main manuscript, however two of these patients were transfusion dependent already and in one the subsequent transfusion was at higher Hb threshold than baseline. As with the rest of this report we are limited by our reliance on clinician documentation. The raw data can give some further insight that might help plan further work.

***Symptom relief is an important outcome measure for this patient group. Whilst I appreciate the authors are reliant on retrospective data it would be useful to know further details on which symptoms had improved if this is available.***

 Agreed, unfortunately, we were unable to answer this in a meaningful way with the documentation available and hopefully this can be analysed further in future work.

***The data presented in Table 3 is helpfully divided by those who did and did not receive simultaneous blood transfusion. It is interesting to see that haemoglobin was higher in the non-transfused group at 6-8 weeks. Can the authors comment on this?***

 We wanted to ensure we presented the results with simultaneous transfusion excluded to ensure effects seen were not just the transfusion in those that received it. As stated, the results are interesting. We are reluctant to comment in more detail within the paper given this analysis was not the aim of the paper, and the sample size becomes very small. Speculating on possible reasons for those individuals, the effect of transfusion in this population is often very transient. It is also possible that those who also required transfusion may have had more multifactorial anaemia and therefore less response to the intravenous iron.

***The discussion is well structured and covers a number of important points. The limitations of the study are well acknowledged with good reference to previous smaller case series. The authors have demonstrated that their model is feasible and may offer patients an alternative option to repeated transfusion, particularly within an ambulatory setting. Steele et al. should be commended on publishing this case series in an area lacking high quality evidence. I would encourage the authors and other palliative care groups to publish future prospective data offering a robust evaluation of the efficacy of intravenous iron against a comparator group.***

 Many thanks again for your time spent reviewing our work.

# Reviewer report: Reviewer response for version 1

**DOI:** 10.21956/amrcopenres.14034.r26623

**Published:** 2021-02-18

**Authors:** Katalin Urban

**Affiliations:** 1 Palliative Care Department, Prince of Wales Hospital, Lismore, NSW, Australia

Thank you for this contribution to the literature in managing iron deficiency in the inpatient palliative care setting, and how national guidelines translate to practice in this situation.

 There is a typo in the abstract, it is reported that 112 cases had iron status evaluated, representing 25/110 of those with mild anaemia, 26/76 with moderate and 41/54 with severe anaemia, however in the results section it is 46/76 for moderate anaemia (not 26) - these numbers add up to 112 whereas those in the abstract do not thus I assume are incorrect, please rectify.  

 The aim and setting are clearly described and the methods section is well written and easy to follow.

 Retrospective data collection often does lead to bias and is dependent on documentation quality - in the Design section, it is stated that the documentation of the presence of classic symptoms of anaemia were sought, however this is very heavily reliant on documentation and an absence of documentation does not necessarily mean they were not present. 

 Prospective collection of data would have added a great deal to the validity of this study, with identification of data to be collected in advance - the authors acknowledge this in limitations.

 With regards to the protocol for investigating iron deficiency anaemia, it would help reproducibility if the authors could clarify how it was decided if oral/iv management would be appropriate - especially as in results it states that 6 patients with AID had clear decisions not to treat on clinical grounds.

 Figure 1 is a great visual representation of the diagnostic process.  All 3 tables are clear and easy to understand.

 Results -  A great overview of the change in practice is provided. Whilst the focus was not outcomes, it is reported that 58% had a documented symptom benefit, however there is no further information on which symptoms and what level of improvement. This information, as well as quality of life measures, would add value. I realise that the aim of this study was not to look at outcomes, and as the authors point out further research is needed to clarify this.

 I am curious why iron levels were not reported (as they are documented in the raw data).

 It is interesting to note, when the raw data is reviewed, that only 2 of the 12 patients treated with iv iron had an MCV over 80 - this is not commented on, though it is discussed that lower MCV does correlate with iron deficiency as expected. I would be interested to hear what the authors make of this. Is this a coincidence?

 A great start to showing that implementation of guidelines is possible and that iron infusions seem doable and safe n the hospice setting. In terms of future directions, a prospective study evaluating the overall benefit to patients would be a great next step.

Is the work clearly and accurately presented and does it cite the current literature?

Yes

If applicable, is the statistical analysis and its interpretation appropriate?

Yes

Are all the source data underlying the results available to ensure full reproducibility?

Yes

Is the study design appropriate and is the work technically sound?

Yes

Are the conclusions drawn adequately supported by the results?

Yes

Are sufficient details of methods and analysis provided to allow replication by others?

Yes

Reviewer Expertise:

palliative care, fatigue, communication, sleep, renal supportive care

I confirm that I have read this submission and believe that I have an appropriate level of expertise to confirm that it is of an acceptable scientific standard.

# Response

**Published:** 2021-02-25

**Authors:** Thomas Steele

**Affiliations:** Marie Curie Cancer Care, UK

Thank you for reviewing our article, your positive feedback and constructive criticisms. We completely agree with your analysis of the limitations of the work, particularly its retrospective design and hope it can be a step towards a prospective study with robust analysis of the effect of iron replacement on symptom burden in future. We have made some amendments based on your comments, which I have explained point by point, along with specific responses to your comments, below.

*Thank you for this contribution to the literature in managing iron deficiency in the inpatient palliative care setting, and how national guidelines translate to practice in this situation.*

*There is a typo in the abstract, it is reported that 112 cases had iron status evaluated, representing 25/110 of those with mild anaemia, 26/76 with moderate and 41/54 with severe anaemia, however in the results section it is 46/76 for moderate anaemia (not 26) - these numbers add up to 112 whereas those in the abstract do not thus I assume are incorrect, please rectify.*

 Thank you for pointing this out, we have amended accordingly.

*The aim and setting are clearly described and the methods section is well written and easy to follow.*

*Retrospective data collection often does lead to bias and is dependent on documentation quality - in the Design section, it is stated that the documentation of the presence of classic symptoms of anaemia were sought, however this is very heavily reliant on documentation and an absence of documentation does not necessarily mean they were not present.*

 Agreed. To make this clearer to the reader we have specified where exactly these were documented (methods section, design) and highlighted reliance on documentation as a limitation.

*Prospective collection of data would have added a great deal to the validity of this study, with identification of data to be collected in advance - the authors acknowledge this in limitations.*

 Hopefully this report will support funding and approvals for a prospective trial.

*With regards to the protocol for investigating iron deficiency anaemia, it would help reproducibility if the authors could clarify how it was decided if oral/iv management would be appropriate - especially as in results it states that 6 patients with AID had clear decisions not to treat on clinical grounds.*

 Thanks for pointing out the need for more clarity here, we have significantly amended the paragraph “protocol for interpretation of iron status and managing iron deficiency” to explain the decision-making process more clearly. Table 2 includes the reason why intravenous iron was indicated (final row).

*Figure 1 is a great visual representation of the diagnostic process.  All 3 tables are clear and easy to understand.*

*Results -  A great overview of the change in practice is provided. Whilst the focus was not outcomes, it is reported that 58% had a documented symptom benefit, however there is no further information on which symptoms and what level of improvement. This information, as well as quality of life measures, would add value. I realise that the aim of this study was not to look at outcomes, and as the authors point out further research is needed to clarify this.*

 Agreed, this was limited by documentation, now more clearly highlighted as a limitation.

*I am curious why iron levels were not reported (as they are documented in the raw data).*

 Iron levels are reported as part of “iron studies” in our locality but aren’t felt to be a reliable indicator of iron status in isolation and ferritin primarily and, in the context of inflammation, transferrin saturation recommended to inform decision making. Ref 11 gives some more detail about the complex area of functional iron deficiency relevant here. In order to make as relevant as possible to clinical practice we therefore reported ferritin/TSAT in the main manuscript but were keen to ensure all parts of iron studies were included in raw data in case of interest to groups in the future.

*It is interesting to note, when the raw data is reviewed, that only 2 of the 12 patients treated with iv iron had an MCV over 80 - this is not commented on, though it is discussed that lower MCV does correlate with iron deficiency as expected. I would be interested to hear what the authors make of this. Is this a coincidence?*

 Thanks for this, it is an interesting observation, especially as in the AID group as a whole the mean MCV is over 80. It is possible that lower MCV could have influenced clinician judgement- in particular the perceived likelihood of iron replacement being helpful. It would be interesting to see this being reported in further studies. Or it could be an observation that would disappear with a larger sample of IV iron patients. A particular area where we need more evidence is functional iron deficiency in which a low MCV wouldn’t typically be seen.

*A great start to showing that implementation of guidelines is possible and that iron infusions seem doable and safe n the hospice setting. In terms of future directions, a prospective study evaluating the overall benefit to patients would be a great next step.*

 Thanks again for the time spent reviewing our work.
